# Lithium reduces blood glucose levels, but aggravates albuminuria in BTBR-*ob/ob* mice

**DOI:** 10.1371/journal.pone.0189485

**Published:** 2017-12-15

**Authors:** Theun de Groot, Lars Damen, Leanne Kosse, Mohammad Alsady, Rosalinda Doty, Ruben Baumgarten, Susan Sheehan, Johan van der Vlag, Ron Korstanje, Peter M. T. Deen

**Affiliations:** 1 The Jackson Laboratory, Bar Harbor, Maine, United States of America; 2 Department of Physiology, Radboud University Medical Center, Nijmegen, The Netherlands; 3 Vivium Care Group, Huizen, The Netherlands; 4 Department of Nephrology, Radboud University Medical Center, Nijmegen, The Netherlands; UCL Institute of Child Health, UNITED KINGDOM

## Abstract

Glycogen synthase kinase 3 (GSK3) plays an important role in the development of diabetes mellitus and renal injury. GSK3 inhibition increases glucose uptake in insulin-insensitive muscle and adipose tissue, while it reduces albuminuria and glomerulosclerosis in acute kidney injury. The effect of chronic GSK3 inhibition in diabetic nephropathy is not known. We tested the effect of lithium, the only clinical GSK3 inhibitor, on the development of diabetes mellitus and kidney injury in a mouse model of diabetic nephropathy. Twelve-week old female BTBR-*ob/ob* mice were treated for 12 weeks with 0, 10 and 40 mmol LiCl/kg after which the development of diabetes and diabetic nephropathy were analysed. In comparison to BTBR-WT mice, *ob/ob* mice demonstrated elevated bodyweight, increased blood glucose/insulin levels, urinary albumin and immunoglobulin G levels, glomerulosclerosis, reduced nephrin abundance and a damaged proximal tubule brush border. The lithium-10 and -40 diets did not affect body weight and resulted in blood lithium levels of respectively <0.25 mM and 0.48 mM. The Li-40 diet fully rescued the elevated non-fasting blood glucose levels. Importantly, glomerular filtration rate was not affected by lithium, while urine albumin and immunoglobulin G content were further elevated. While lithium did not worsen the glomerulosclerosis, proximal tubule function seemed affected by lithium, as urinary NGAL levels were significantly increased. These results demonstrate that lithium attenuates non-fasting blood glucose levels in diabetic mice, but aggravates urinary albumin and immunoglobulin G content, possibly resulting from proximal tubule dysfunction.

## Introduction

Diabetes mellitus (DM) often leads to diabetic nephropathy, a progressive decline in kidney function. Diabetic nephropathy starts with hypertrophy of renal proximal tubules and hyperfiltration.[1] Release of growth factors, cytokines and pro-inflammatory markers consequently leads to oxidative stress, manifesting as tubular atrophy and interstitial fibrosis.[2–4] A silent phase follows, as the glomerular filtration rate (GFR) turns back to normal.[5] After this period, a progressive decline in GFR, and thus kidney function, develops.[6, 7] This decline in renal function is histopathologically characterized by a substantial thickened glomerular basement membrane (GBM), increased diffuse or nodular glomerulosclerosis, hyaline arteriosclerosis, tubulointerstitial fibrosis and tubulointerstitial atrophy.[8] Diabetic nephropathy is diagnosed via standard criteria for chronic kidney disease (CKD): a GFR below 60 mL/min/1.73m^2^ and/or more than 30mg albumin/g creatinine in urine.^[^^9^^–^^11^^]^ The high impact of diabetes as a cause for kidney disease is illustrated by the fact that 25–40% of all patients who have DM for 20–25 years eventually develop CKD.[12] Due to the large incidence of DM, ~43% of all patients with end-stage renal disease is caused by DM and this is expected to increase, as the World Health Organization recently reported that the prevalence of diabetes quadrupled between 1980 and 2014.[10, 13, 14]

The serine/tyrosine kinase glycogen synthase kinase 3 (GSK3) has been shown to play a prominent role in cellular glucose uptake and storage. In healthy individuals, glucose uptake is stimulated by binding of insulin to its receptor, as this activates the insulin receptor substrate-1 (IRS-1), PI3-kinase and Akt, which subsequently enhances the plasma membrane abundance of glucose transporter type 4 (GLUT4) therewith increasing cellular glucose uptake.[15, 16] Glucose storage is also activated by Akt, as it stimulates glycogen synthase activity leading to intracellular glucose storage in the form of glycogen.[17, 18] As GSK3 inhibits both IRS-1 and glycogen synthase, it attenuates cellular glucose uptake and glycogen storage.[15, 16, 19, 20] As a consequence, it has been thought that GSK3 inhibition could be beneficial in DM. Indeed, GSK3 activity/abundance was elevated in various tissues of diabetic animals and patients[21–23] and GSK3 inhibition or haplo-insufficiency reduced insulin resistance and blood glucose levels in diabetic mice.[24–26]

Interestingly, recent studies indicated that moderate GSK3 inhibition is also beneficial in the prevention and treatment of acute kidney injury (AKI). Firstly, inhibition of GSK3β in murine models of cisplatin- and ischemia/reperfusion-induced AKI boosted cell proliferation and the repair machinery of renal tubular epithelia, which coincided with increased levels of cyclinD1, c-Myc and HIF-1α.[27, 28] Secondly, GSK3 inhibition in LPS or Adriamycin-induced AKI decreased pro-inflammatory signalling and attenuated glomerular damage.[29] Lastly, GSK3 inhibitors decreased ROS-dependent apoptosis of renal epithelial cells in rodents with paraquat-induced AKI.[30] In agreement with these findings, mice overexpressing a GSK3 variant, mediating resistance to inhibition by Akt, demonstrated albuminuria and glomerulosclerosis.[31] Altogether, these data indicated that GSK3 inhibition attenuates proximal tubule and glomerular damage.

Considering the beneficial effects of GSK3 inhibition in DM and renal injury, we hypothesized that GSK3 inhibition may attenuate development of diabetic nephropathy. The only clinically available GSK3 inhibitor is lithium, which inhibits GSK3 in a direct and indirect manner, by respectively acting as a competitor for Mg^2+^ binding,[23] which is required for substrate phosphorylation, and by promoting its phosphorylation of GSK3α and GSK3β at serine 21 or 9, respectively, resulting in their inactivation.^[^^20^^]^ In clinics, lithium is used to treat patients with bipolar disorder in order to prevent episodes of mania using a daily dose of 900–1800 mg.[27, 32] It has been shown that this lithium dose leads in some patients to the development of kidney damage.[27, 33] Importantly, however, Hu et al demonstrated that a subclinical daily dose of 100 mg lithium already suffices to reduce non-fasting blood glucose levels in diabetic patients.[34]

Therefore, we here investigated the effect of subclinical lithium doses on the development of diabetic nephropathy. For this, we used BTBR-*ob/ob* mice, a widely-accepted DM type 2 (DM2) model, as, due to a homozygous *ob/ob* mutation inducing leptin deficiency, these mice consume excessive amount of food, and develop obesity and diabetes. Importantly, the BTBR-*ob/ob* mice develop signs of both early and advanced human diabetic nephropathy, as they exhibit albuminuria at an age of 4 weeks and morphological glomerular lesions at an age of 8 weeks.[11]

## Materials and methods

### Animal model

Twelve-week old female wild-type and *ob/ob* BTBR *T*^*+*^ *Itpr3*^*tf*^/J (BTBR) mice were obtained from The Jackson Laboratory. Mice were housed at The Jackson Laboratory in a temperature-controlled room with a 12:12h light-dark cycle and had *ad libitum* access to water and food (standard chow (7013, NIH-31 Modified) or standard chow supplemented with respectively 10 and 40 mmol lithiumchloride (LiCl)/kg chow, Harlan Laboratories Inc, Madison, WI, USA). Immediately before sacrifice by cervical dislocation, which took place a few hours after initiation of the light cycle, blood was collected via submandibular bleeding. Kidneys were collected and processed for histology or immunoblotting, as described.[35] Animal studies were approved by The Jackson Laboratory's Institutional Animal Care and Use Committee.

### GFR determination

GFR was determined using the FITC-Inulin clearance method.[36] At week 11, mice received a single bolus injection of dialysed FITC-inulin. Before FITC-inulin injection, blood was collected to determine baseline fluorescence. At 3, 5, 7, 10, 15, 35, 56, and 75 minutes after injection, blood was collected via the tail. Blood fluorescence was determined using a Cytofluor II Fluorescence Multiwell Plate Reader (PerSeptive Biosystems) with 485nm excitation and 538nm emission. Renal excretion rate of inulin was determined based on the decay fluorescence counts over time. Based on this excretion rate, the GFR was determined.

### Glucose tolerance test

At 10 weeks, a glucose tolerance test was performed. After a 4-hour fast, mice received an intraperitoneal injection of 2 mg/g body weight glucose. Before and at 15, 30, 60 and 120 minutes after injection, blood was collected from the tail and glucose levels were immediately determined with a blood glucose meter (OneTouch Ultra^®^2). Before sacrifice, blood was collected by submandibular bleeding and non-fasting glucose levels were immediately determined using the blood glucose meter.

### Blood and urine analysis

Blood was centrifuged at 3000xg for 5 minutes to separate serum from blood cells. Serum concentration of lithium was measured using The Medimate Mini-Lab (Enschede, The Netherlands). Insulin concentration in serum was measured using Ultra Sensitive Insulin ELISA (Cristal Chemical Inc.). Additional serum analyses of blood urea nitrogen (BUN) and neutrophil gelatinase associated lipocalin-2 (NGAL) were performed using the NCal NIST-Calibrated Kit (ARBOR ASSAYS) and Mouse-NGAL DuoSet Kit (RnD Systems) respectively. Urine was analysed for creatinine and albumin on a Beckman Synchron CX5 Chemistry Analyzer (Fullerton, CA, USA) by the Histology department of The Jackson Laboratory, as described[37], while urinary IgG levels were determined with the Mouse-IgG ELISA (Roche, V.10), and NAG-activity with NAG Activity Assay Kit (Abcam).

### Histology

Kidney were isolated and stored in 3.5% PFA solution. After fixation for two days, kidneys were embedded in paraffin. Kidney slides were stained with Periodic acid-Schiff or Picro Sirius Red. Assessment of the slides was performed using blind randomization.

### Immunoblotting

Immunoblotting was performed as described[38]. Primary antibodies included rabbit anti-cubilin and sheep anti-megalin (gifts from Prof. Pierre Verroust, INSERM, Paris, France), mouse anti-GSK3β (#610201, BD Biosciences), rabbit anti-pGSK3β (#9336S, Cell Signalling Technology) and guinea pig anti-nephrin (#20R-NP002, Fitzgerald Industries International). Proteins were visualized using enhanced chemiluminescence (Pierce) and the “ChemiDoc XRS” (Bio-Rad). For semi-quantification the QuantityOne software (Bio-Rad) was used, while Coomassie blue staining was used to determine and correct for total protein input.

### RNA isolation, primer design and reverse transcription-quantitative PCR (RT-qPCR)

RNA from renal cortex was isolated using TRIzol® (Gibco Life Technologies, Rockville, MD, USA) and 1.5 μg of RNA was reverse transcribed using the Moloney murine leukemia virus reverse transcriptase kit (Promega, Madison, WI, USA), according to the suppliers’ protocol. Following a 10-fold dilution, mRNA levels were assessed by RT-qPCR using SYBR-green (Applied Biosystems, Foster City, CA, USA) and normalized to housekeeping gene *Rplp0* (36B4). Primer sequences were as listed in S1 Table.

### Statistical analysis

All statistical analyses were performed in GraphPad Prism. Statistical analyses were performed using one-way ANOVA with Bonferroni correction. Differences were considered significant at p<0.05. Data are presented as means and SEMs.

## Results

### GSK3 activity is increased in kidneys of ob/ob mice

**Fig 1:**
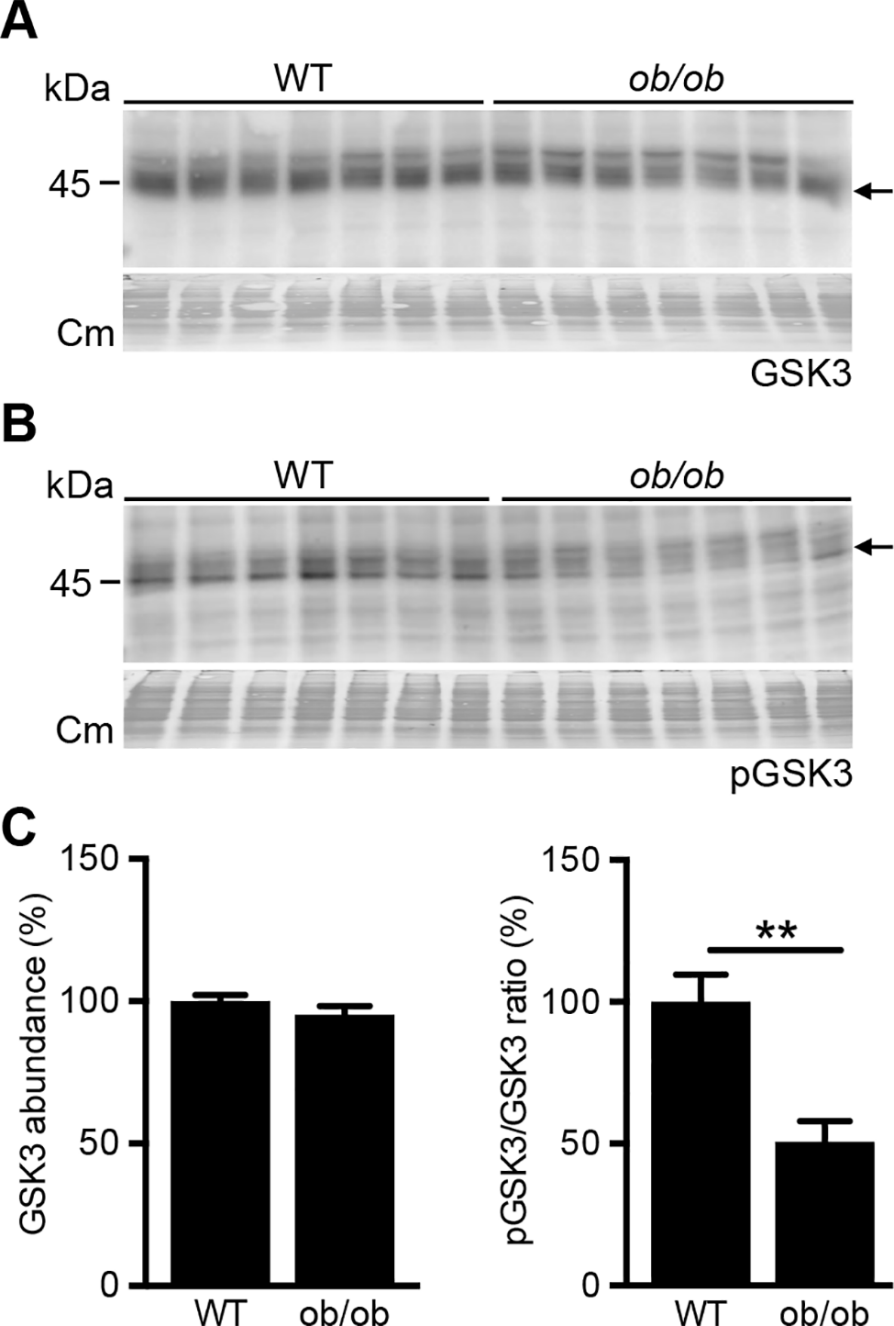
pGSK3/GSK3 ratio in BTBR-WT and *ob/ob* mice. Kidneys from 24-week old female BTBR-WT and -*ob/ob* mice were isolated and protein lysates were immunoblotted for pGSK3 and GSK3. Representative immunoblots for WT and *ob/ob* mice for (A) GSK3 and (B) pGSK3 are depicted. Corresponding densitometric analysis of (C) total GSK3 abundance and (D) pGSK3/GSK3 ratio (n = 9 per group). **p<0.01. Cm, Coomassie.

Diabetic animals and patients demonstrated an increased abundance or decreased phosphorylation of GSK, indicative of an increased activity.[21–23] As overexpression of active GSK3 caused albuminuria and glomerulosclerosis,[31] we first wanted to establish whether GSK3 activity was altered in 24-week old BTBR-*ob/ob* mice, as at this age these mice demonstrate diabetes, albuminuria and glomerular damage.[11] Using whole kidney homogenates of 24 week-old BTBR-wild type (WT) and BTBR-*ob/ob* mice, total and phosphorylated GSK3 abundance was examined by immunoblotting (Fig 1A and 1B). Semi-quantification demonstrated that total GSK3 abundance was not different between both groups, but that the pGSK3/GSK3 ratio was significantly decreased in BTBR-*ob/ob* mice, suggesting that diabetes caused GSK3 overactivity in the kidneys of these mice (Fig 1C).

### Lithium reduces non-fasting blood glucose levels in ob/ob mice

To determine whether the GSK3 inhibitor lithium might constitute a treatment for diabetic nephropathy, we wanted to test the effect of lithium at a point in which early signs of diabetic nephropathy were already observed. In line with data from others,[11] our 12 weeks old BTBR-*ob/ob* mice demonstrated an increased body weight (53.6 vs 28.1 g, p<0.05) and elevated albumin creatinine ratio (ACR) as compared to WT mice (101.8 vs 12.0 mg/g, p<0.05). After randomizing for body weight and ACR, the *ob/ob* BTBR mice received standard chow (control) or chow supplemented with 10 (Li-10) or 40 (Li-40) mmol LiCl per kg food for 12 weeks (Table 1). As a control for obesity/DM2 development in *ob/ob* BTBR mice, an additional group of WT mice only received standard chow. The Li-10 and -40 diets did not affect body weight and led to serum lithium levels of <0.25 mM and 0.48 ± 0.04 mM, respectively (Table 1). These levels are subclinical as in bipolar patients a range of 0.6–1.0 mM is maintained.

**Table 1:** Body weight and lithium levels.

Group	Bodyweight (g)			Lithium (mM)
	Week 0	Week 6	Week 12	Week 12
**WT**	28.1 ± 0.6	32.3 ± 0.9	35.5 ± 1.3	ND
***ob/ob***	53.6 ± 1.3***	73.4 ± 1.3***	83.5 ± 2.4***	ND
***ob/ob* Li-10**	54.3 ± 1.8	71.7 ± 2.0	79.2 ± 3.0	<0.25
***ob/ob* Li-40**	53.8 ± 1.5	72.4 ± 1.9	78.7 ±2.0	0.48 ± 0.04

12-week old female BTBR-WT and -*ob/ob* mice received standard chow or chow with lithium supplementation (10 or 40 LiCl/kg) for 12 weeks. Lower detection range of the lithium analyser was 0.25 mM.

***p<0.001 vs. WT.

ND, not determined.

**Fig 2:**
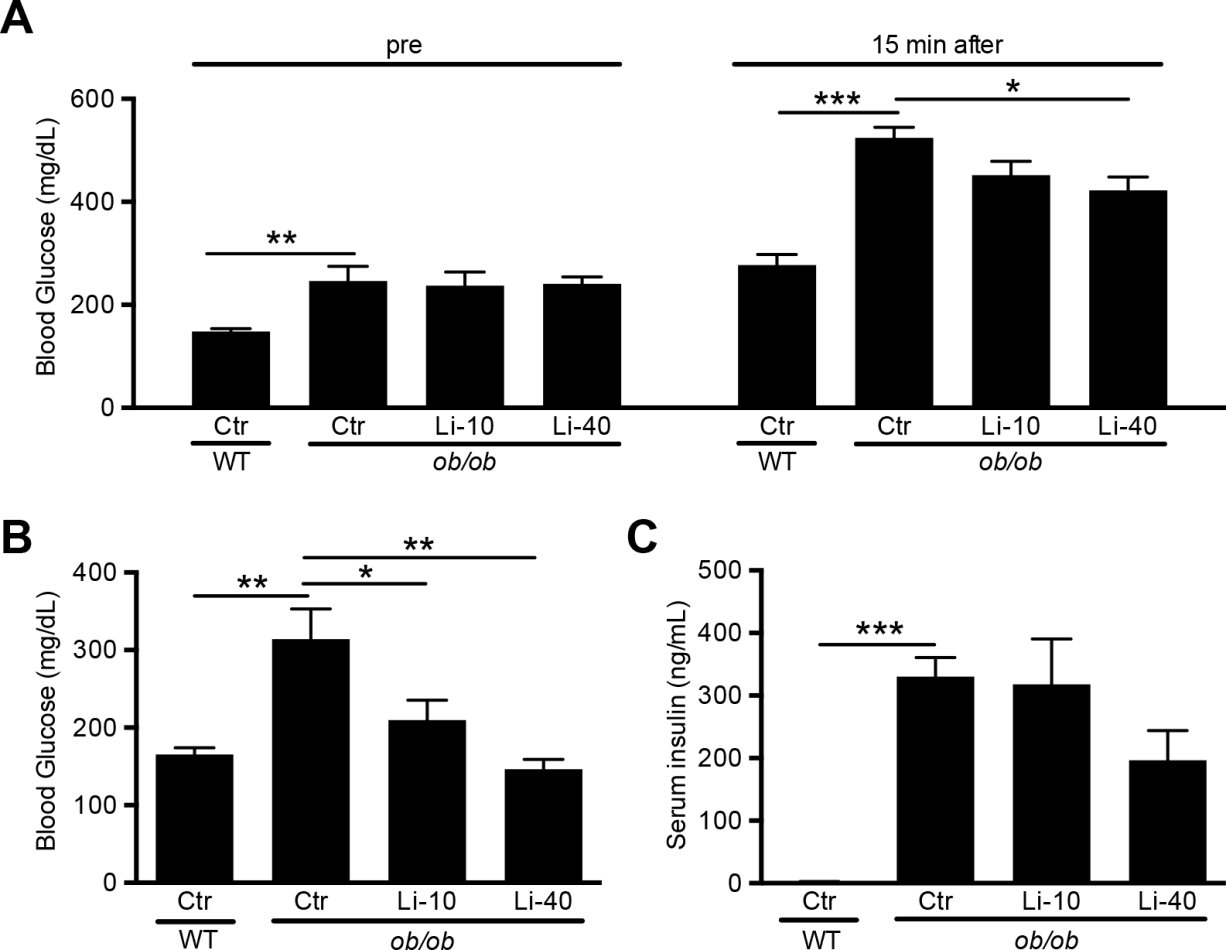
The effect of lithium on blood glucose and insulin levels. 12-week old female BTBR-WT and -*ob/ob* mice received standard chow or chow with lithium supplementation (10 or 40 mmol LiCl/kg) for 12 weeks. Two weeks before sacrifice, mice were fasted for 4 hours, after which they received an intraperitoneal injection of 2 mg glucose/g bodyweight. Blood was collected before and 15 minutes after injection and blood glucose levels were determined (A). At the time of sacrifice blood was collected to analyze non-fasting (B) glucose and (C) insulin levels, n = 9 per group. *p<0.05; **p<0.01; ***p<0.001.

To determine the effect of lithium on DM2 itself, a glucose tolerance test was performed after 10 weeks of lithium treatment. Following fasting for 4 hours, blood glucose levels were increased in the *ob/ob* mice, which were not affected by lithium (Fig 2A). Then, following an intraperitoneal glucose injection, the mice were subjected to a glucose tolerance test. At 15 minutes after injection, blood glucose levels were strongly elevated in the *ob/ob* mice as compared to WT controls, while lithium attenuated this elevation (Fig 2A). Notably, blood glucose levels in *ob/ob* mice at 30, 60 and 120 minutes were mostly above the upper detection limit of the glucose meter (S2 Table), precluding further information. At the time of sacrifice, non-fasting blood glucose levels were determined. *Ob/ob* mice also exhibited elevated non-fasting blood glucose, which was reduced in the lithium-treated mice (Fig 2B). In fact, blood glucose levels of the *ob/ob* mice on Li-40 diet were similar to those of the WT controls. In line with an increased glucose uptake and tolerance with GSK inhibition, the non-fasting insulin levels were elevated in *ob/ob* mice as compared to WT mice, and tended to decrease (p = 0.14) in the mice on the Li-40 diet (Fig 2C).

### Lithium increases urinary albumin and IgG content in BTBR-ob/ob mice

**Fig 3:**
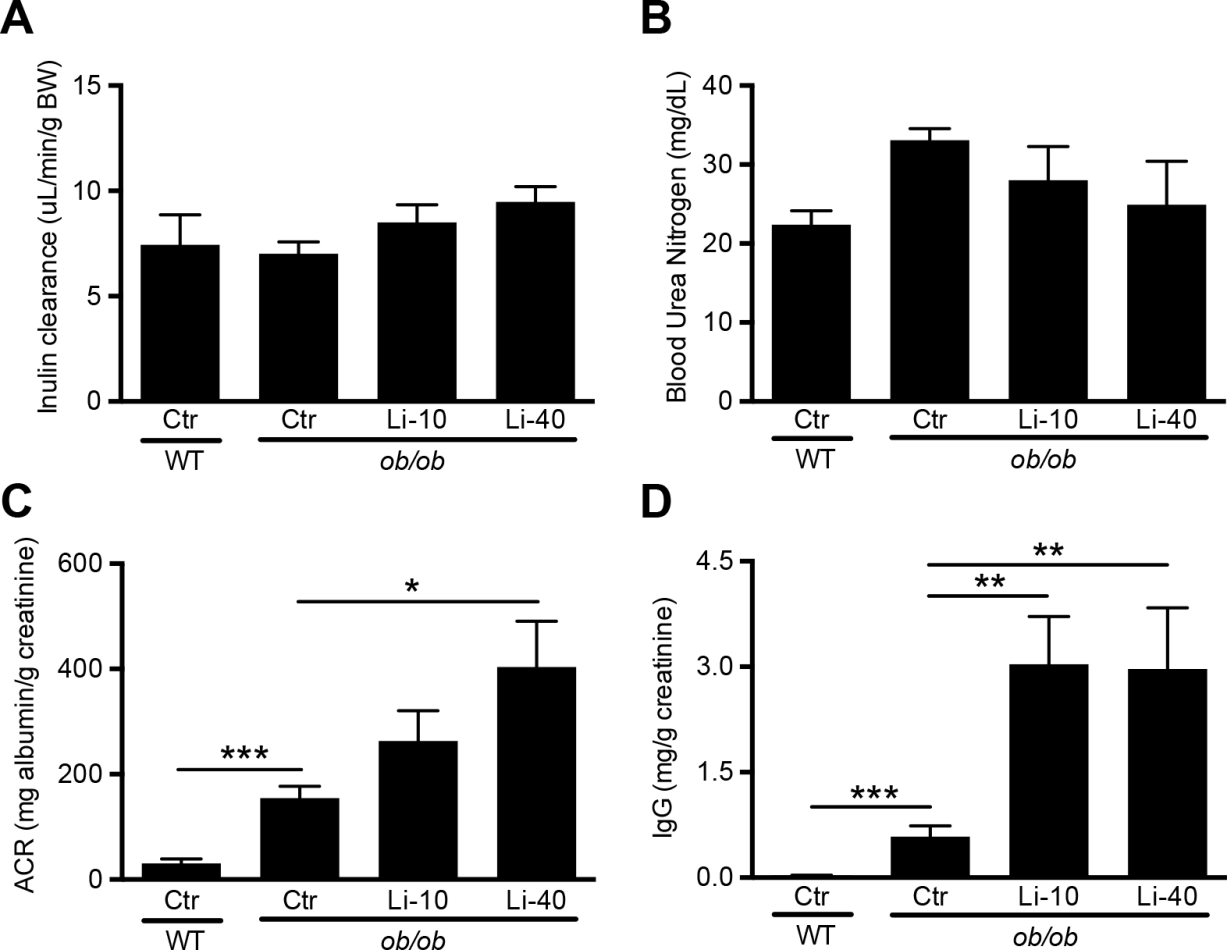
The effect of lithium on GFR, BUN, and urinary albumin and IgG levels. 12-week old female BTBR-WT and -ob/ob mice received standard chow or chow with lithium supplementation (10 or 40 mmol LiCl/kg) for 12 weeks. After 11 weeks of treatment, mice received a FITC-inulin bolus injection. Inulin clearance was determined via the decay of FITC-inulin in blood (A). At the time of sacrifice, blood was isolated and BUN was determined (B), while spot urine was collected earlier that week to determine (C) albumin-creatinine ratio and (D) IgG content, n = 9 per group. *p<0.05; ***p<0.001; ACR, albumin-creatinine ratio.

To establish the effect of lithium on diabetic nephropathy, we analysed the mice for GFR by inulin clearance, blood urea nitrogen (BUN) and ACR. After correction for bodyweight, thereby correcting for differences in kidney size,[39] GFR was not statistically different between the four groups (Fig 3A). There was also no significant difference in BUN between the groups, although it tended to increase in the *ob/ob* mice (p = 0.15) and tended to attenuate again with lithium (p = 0.42) (Fig 3B). ACR, determined from spot urine isolated at 12 weeks of treatment, was elevated in *ob/ob*-Ctr mice compared to WT mice. Importantly, lithium treatment increased ACR, which was significant in the Li-40 diet group (Fig 3C). Finally, spot urine was also analysed for the presence of immunoglobulin G (IgG), which is only found in urine when the filtration barrier is damaged.[40, 41] Urinary IgG levels were significantly increased in *ob/ob* mice as compared to WT controls, which were aggravated by both the Li-10 and Li-40 diet (Fig 3D).

### Lithium-induced aggravation of albuminuria is likely not caused by glomerular damage

**Fig 4:**
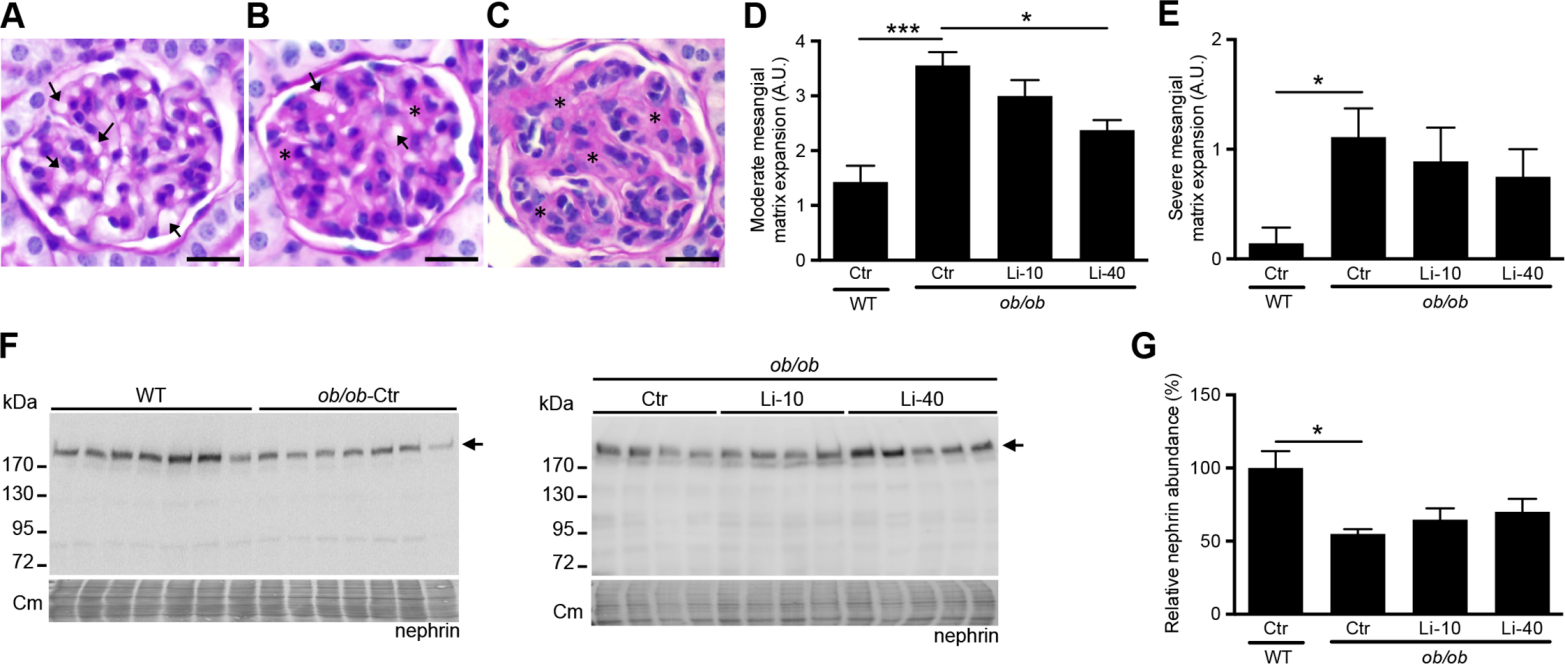
Analysis of glomerular injury upon lithium treatment. 12-week old female BTBR-WT and -*ob/ob* mice received standard chow or chow with lithium supplementation (10 or 40 LiCl mmol/kg) for 12 weeks. After sacrifice, kidneys were isolated and the absence (A) or presence of moderate (B) or severe (C) mesangial matrix expansion was determined using PAS staining. Moderate and severe mesangial matrix expansion was defined as an increased presence of mesangial matrix (indicated by stars) with the moderate form still having some open capillary lumens (indicated by arrows) while these are basically absent in the severe form. Scale bar indicates 10 μM. The percentage of glomeruli with moderate or severe mesangial matrix expansion was scored as follows: 0) 0–1%, 1) 1–5%, 2) 5–20%, 3) 20–50%, 4) >50% (D and E), n = 9 per group. Kidney lysates were used to determine nephrin abundance via immunoblotting (F) and subsequent semi-quantification (G), n = 9 per group. *p<0.05; ***p<0.001. Cm, Coomassie.

Both albumin and IgG levels are elevated in the urine of *ob/ob*-Ctr mice as compared to WT mice, indicating that the glomerular filtration barrier is damaged in the obese mice. The further elevation of urinary albumin and IgG levels upon lithium treatment might be due to further deterioration of glomerular function, but might also result from injury to the proximal tubule, as both albumin and IgG are reabsorbed by the proximal tubules following filtration leakage.[42] To investigate the origin of the increased urinary albumin and IgG levels in the lithium treated *ob/ob* mice, we first assessed the extent of mesangial matrix expansion using PAS staining (Fig 4A–4C). In line with the elevated urinary albumin and IgG levels, *ob/ob*-Ctr mice demonstrated significantly more glomeruli with moderate and severe mesangial matrix expansion as compared to WT mice (Fig 4D and 4E). Interestingly, the number of moderately-affected glomeruli was reduced in the Li-40 group, while no significant differences were found with the other groups (Fig 4D and 4E). To obtain additional information on the abundance of podocytes, which form the filtration barrier, we next analysed our kidney samples for mRNA and protein levels of proteins that are characteristic for podocytes. However, podocin, nephrin, synaptopodin and podoplanin mRNAs levels were not affected by lithium (S1 Fig). Moreover, immunoblotting revealed that the abundance of nephrin was significantly reduced in the *ob/ob*-Ctr mice as compared to WT mice, which seemed to reverse in the Li-40 group, but this was not significant (p = 0.33) (Fig 4F and 4G). The nephrin immunoblotting was specific, because only one band around the predicted mass (180 kDa) was detected, which was observed in samples derived from the cortex, but not medulla (S2 Fig).

### Lithium may affect proximal tubular function

**Fig 5:**
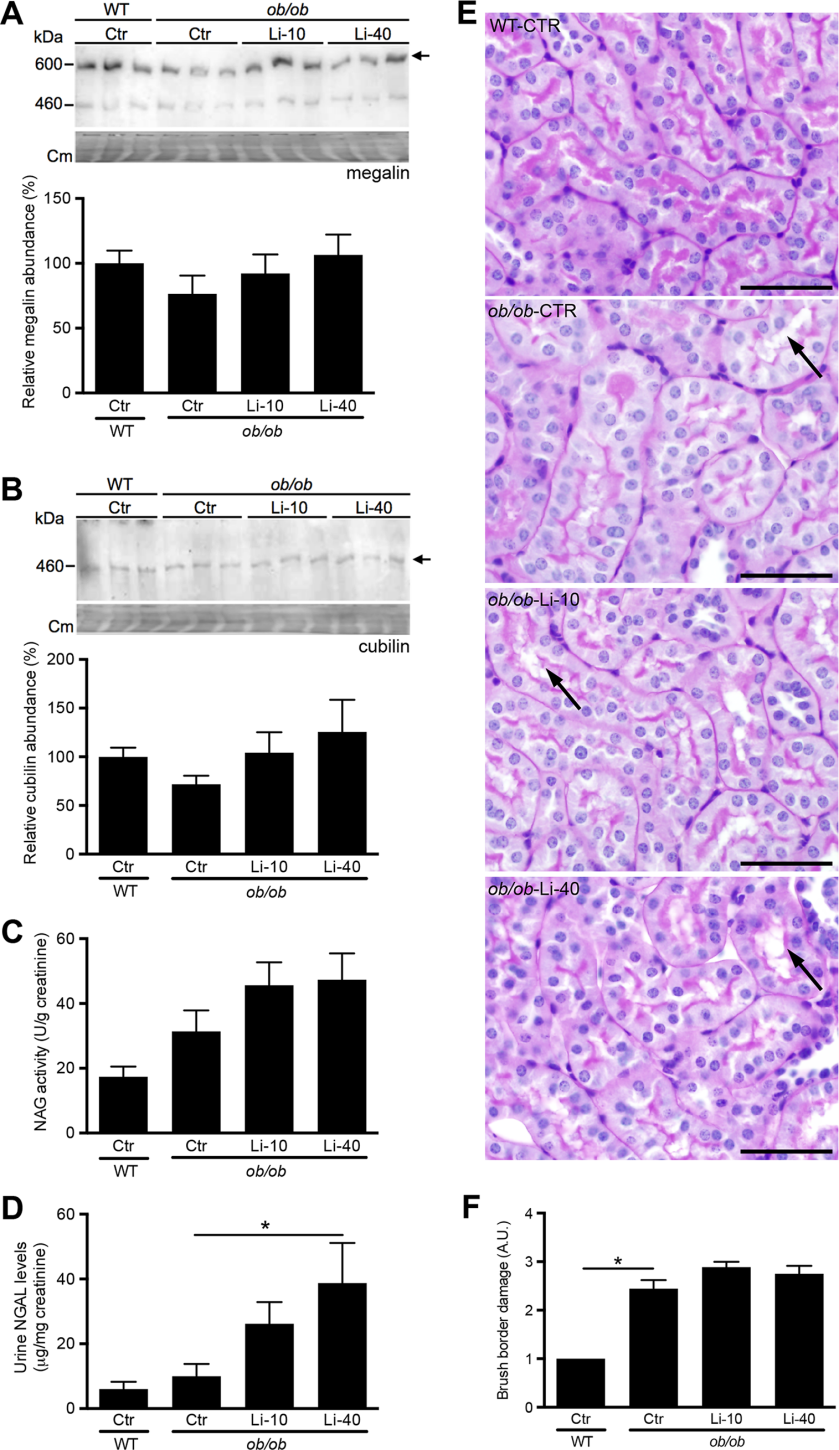
Analysis of proximal tubule injury upon lithium treatment. 12-week old female BTBR-WT and -*ob/ob* mice received standard chow or chow with lithium supplementation (10 or 40 LiCl/kg) for 12 weeks. Kidney lysates were used to immunoblot for megalin (A) and cubilin (B) abundance, as shown by representative images in the upper panels, and subsequent densitometric analysis in lower panels (n = 9/group). Arrows indicate the 600-kDa bands for megalin and 460-kDa band for cubilin. Spot urine, collected in the week before sacrifice, was used to determine NAG activity (C) and NGAL (D) levels. Kidney sections were stained with PAS to determine brush border damage in the different groups, as is shown in the representative pictures (E). The scale bar represents 50 μM. The presence of brush border damage was scored as follows: 0) none, 1) minimal, 2) mild, 3) moderate and 4) severe (F). *p<0.05; ***p<0.001. Cm, Coomassie.

**Fig 6:**
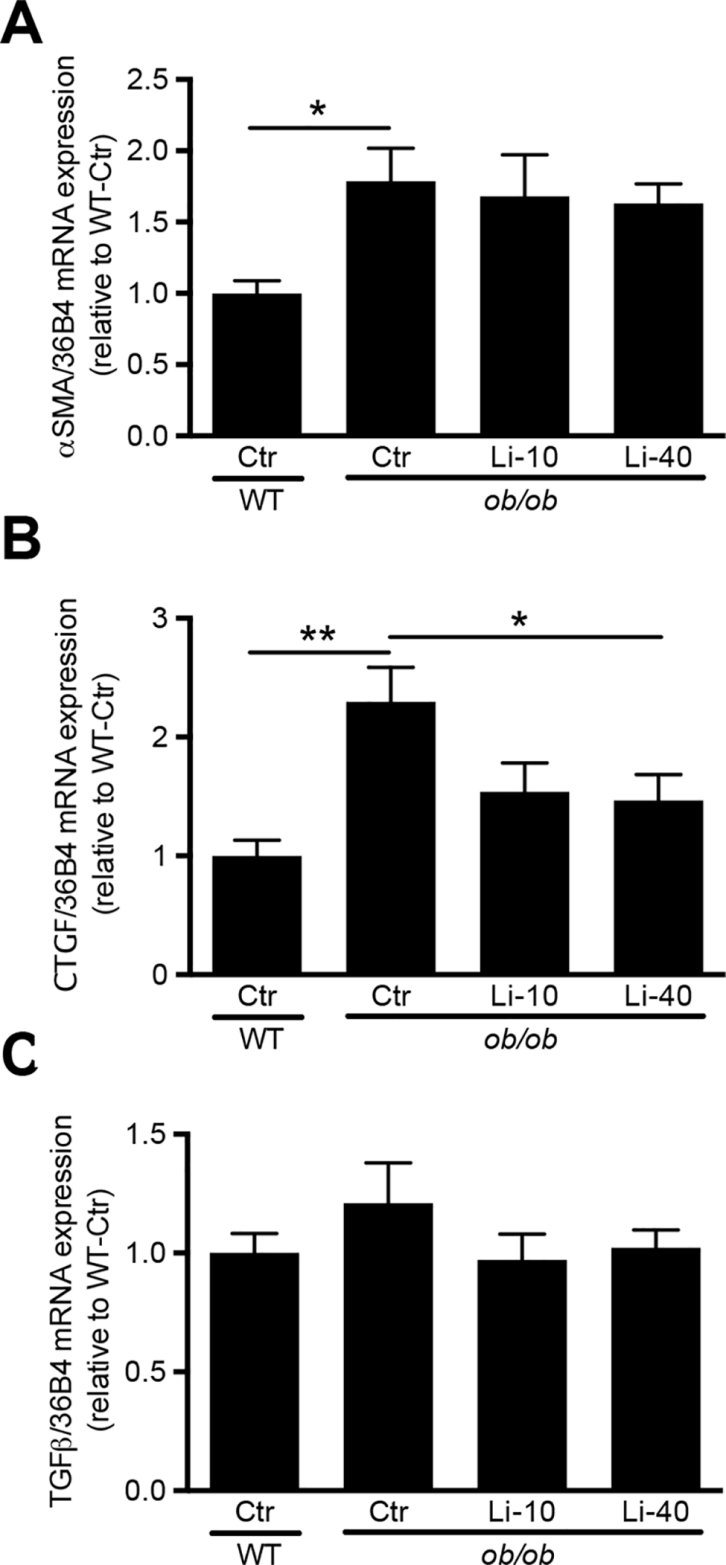
The effect of lithium on cortical αSMA, CTGF and TGFβ mRNA levels. 12-week old female BTBR-WT and -*ob/ob* mice received standard chow or chow with lithium supplementation (10 or 40 LiCl/kg) for 12 weeks. After RNA isolation from cortex, mRNA levels of (A) αSMA, (B) CTGF and (C) TGFβ were determined by qPCR using 36B4 as a housekeeping gene. *p<0.05; **p<0.01.

As proximal tubules reabsorb both albumin and IgG[43] in case of a damaged renal filter, we also tested whether dysfunction of this segment due to lithium may have caused the elevated urinary albumin and IgG levels observed with lithium. However, immunoblotting for megalin and cubilin, which are essential for albumin reabsorption in proximal tubules, revealed no differences between control and lithium-treated *ob/ob* mice (Fig 5A and 5B). To further investigate potential lithium-induced damage to the proximal tubule, we analysed the urinary activity and abundance of two different proximal tubule damage markers, being N-Acetylglucosaminidase (NAG) and serum neutrophil gelatinase associated lipocalin-2 (NGAL), respectively. While urinary NAG activity was not different between the groups (Fig 5C), urinary NGAL levels were significantly elevated by lithium (Fig 5D). Finally, the proximal brush border was stained using periodic acid-Schiff (PAS), as its disappearance indicates proximal tubule damage, as also found in early stages of diabetic nephropathy[44, 45]. In *ob/ob* mice the number of renal tubular epithelia with a reduced PAS staining in the brush border was increased as compared to WT mice, which was not significantly affected by lithium (Fig 5E and 5F). Finally, we performed some additional analyses to better understand the effects of lithium application in diabetic nephropathy. Using Picro Sirius Red staining, interstitial fibrosis was scored as absent, minimal or mild (S3A–S3C Fig). No differences were found between groups (S3D Fig). Furthermore, cortical mRNA levels of αSMA, CTGF, TGFβ and different inflammation markers were assessed. While no significant differences were found for the various inflammatory markers (S4 Fig), lithium partially reversed CTGF mRNA levels, but did not significantly reduce the increased αSMA levels in the ob/ob-Ctr mice, as compared to WT (Fig 6). No significant differences were found in TGFβ mRNA levels (Fig 6).

## Discussion

### Lithium reduces blood glucose levels via mechanisms likely involving insulin signaling

This study demonstrated that low lithium doses attenuate non-fasting blood glucose in BTBR-*ob/ob* mice, while fasting blood glucose was not affected. These low lithium doses (10 and 40 mmol/kg food) resulted in blood lithium levels of respectively <0.25 mM and 0.48 mM, which are both below the clinically used blood lithium concentration used to treat bipolar patients (0.6–1.0 mM). Interestingly, daily administration of 100 mg of lithium carbonate in diabetic patients, which is a ~10-fold lower dose as normally administered to bipolar patients, also caused a reduction in postprandial blood glucose levels, while fasting blood glucose levels were not affected.[34] In agreement, other studies demonstrate that the beneficial effect of lithium on blood glucose handling only occurs in presence of insulin.[34, 46] The precise mechanism by which lithium reduces blood glucose handling was not investigated in these studies although it likely involves its effect on GSK3 activity and subsequent regulation of IRS-1 and glycogen synthase. Thus, lithium likely enhances the efficacy of insulin during insulin resistance.[46, 47] This lithium-insulin connection might explain why in our mouse study and in these diabetic patients lithium decreases blood glucose levels only in non-fasting conditions. In the latter situation, insulin levels are very low, as blood glucose levels are maintained via glycogenolysis, in which glucose is formed from glycogen.[48]

### Lithium does not affect GFR in BTBR-ob/ob mice

To determine the effect of lithium on kidney injury in our diabetic mice, we first measured GFR, as this is also used to diagnose diabetic nephropathy in patients. For this, we assessed FITC-inulin clearance, as creatinine clearance is not a valid marker for GFR in mice.[49] Strikingly, the GFR of 24-week-old BTBR-*ob/ob* mice was similar to WT mice, despite overt glomerulosclerosis and albuminuria in the *ob/ob* mice. Also, both lithium diets did not affect GFR. It must be noted though, that GFR was calculated as inulin clearance per gram body weight, although other studies report GFR without correcting for body weight or by correcting for kidney weight.[36, 50] As kidney weight was not determined by us, but has previously been shown to be increased by ~2-fold in BTBR-*ob/ob* mice as compared to WT mice,[39] we choose to correct for bodyweight, which was increased by 2.3 fold. Moreover, BUN levels were not statistically different between the groups but tended to increase in the *ob/ob* mice as compared to WT mice. These findings are in agreement with our GFR correction for bodyweight, as in comparison to WT mice, the *ob/ob* mice would have a hyperfiltration if not corrected for body weight, which would not be in agreement with their BUN levels. Altogether, it is likely that GFR is not yet affected in 24-week-old female BTBR-*ob/ob* mice, suggesting that this model represents a relative early stage of diabetic nephropathy in which a pronounced GFR decline is not yet present.

### Lithium aggravates urinary albumin and IgG content in mice with diabetic nephropathy

As found previously, BTBR-*ob/ob* mice exhibited elevated urine albumin levels as compared to WT BTBR mice.[11] Additionally, we identified that the urine of the *ob/ob* mice contained significantly higher levels of IgG, indicating glomerular damage, as IgG is normally not filtered by the glomerulus.[40, 41] Lithium further aggravated the urinary content of albumin and IgG. As both albumin and IgG are normally reabsorbed in the proximal tubule in case of filtration damage, this aggravation can result from deterioration of proximal tubule or glomerular function.[42] The latter did not seem affected, as the enhanced mesangial matrix expansion in the BTBR-*ob/ob* mice was decreased by lithium and the reduced nephrin abundance in the *ob/ob* mice was not further reduced by lithium, but even tended to be attenuated. Furthermore, mRNA levels of the typical podocyte proteins nephrin, podocin, podoplanin and synaptopodin were unchanged with lithium. Despite the absence of any indication of lithium-induced glomerular injury, we also did not yield strong evidence that lithium caused proximal tubule injury, as the protein abundance of megalin and cubilin, the receptors responsible for albumin uptake,[42] were not affected by lithium treatment. Moreover, NAG activity, a marker for proximal tubule injury, was also not significantly affected by lithium, although it tended to increase. Only urinary NGAL content was significantly elevated by lithium. Altogether, there is no strong evidence that lithium caused glomerular injury. As proximal tubule seemed to exhibit a little more damage in the lithium groups, the increased urinary albumin and IgG content most likely originates from proximal tubule injury. Although current results are not sufficient to establish the location of damage, a lithium-induced injury of proximal tubules would be more in line with data obtained in studies analysing the renotoxic effects of clinical lithium administration. Lithium-treated bipolar patients with CKD are characterized by the presence of tubular atrophy and interstitial fibrosis, while only a smaller part demonstrates glomerulosclerosis.[51] Also in rabbits it has been shown that lithium first causes tubular atrophy and interstitial fibrosis, while glomerulosclerosis develops much later in disease.[52] Altogether, our data indicate that administration of subclinical lithium dosi is likely not a good strategy to combat diabetic nephropathy.

### Molecular mechanism underlying lithium-induced albuminuria remains elusive

We found that lithium increased urine albumin and IgG levels, however the molecular mechanism behind these observations was not identified. Most likely these effects of lithium involve GSK3 inhibition. It is known that GKS3 plays an important role in the function of proximal tubules and podocytes,[53, 54] the most likely origins for albuminuria. By inhibiting GSK3, lithium might have directly affected the function of these cells. We found however that lithium did not increase GSK3 phosphorylation, an inhibitory modulation, in whole kidney lysates (**S5 Fig**). As such, lithium-induced albuminuria is most likely due to the direct inhibition of GSK3 by lithium via its competition for magnesium[23]. Alternatively, the increased urine albumin and IgG levels might be the consequence of the effects of lithium on blood glucose or insulin levels. While altered blood glucose levels affect both the glomerulus and proximal tubules, altered insulin levels would mainly affect podocyte function, as insulin receptors are present on these cells and were shown to play a role in filtration.[39] A role for direct GSK3 inhibition is however more likely as reduced blood glucose and insulin levels, as observed in this study, would rather lead to a reduction in albuminuria.[39] Future studies should aim to elucidate the molecular effects of lithium by analyzing the molecular effects in specific cell types of the kidney.

### The application of lithium in bipolar patients with diabetes mellitus

Thus, although lithium is not likely to become a treatment for diabetic nephropathy, there are thousands of bipolar patients with DM who are treated with lithium for their bipolar disease. The precise effect of such higher lithium dose on kidney function in this subpopulation is not known, however a recent large retrospective analysis did not find an increased risk of CKD, determined by a decrease in eGFR, in lithium-using patients with DM as compared with lithium only.[55] However, the number of DM patients was rather small. Therefore, it would be worthwhile to compare renal decline in DM patients who are treated with lithium with those who are treated with other treatments for mental disease.

## Supporting information

S1 Fig The effect of lithium on mRNA levels of nephrin, podocin, podoplanin and synaptopodin. 12-week old female BTBR-WT and -*ob/ob* mice received standard chow or chow with lithium supplementation (10 or 40 LiCl/kg) for 12 weeks. After RNA isolation from cortex, mRNA levels of nephrin, podocin, podoplanin and synaptopodin were determined by qPCR using 36B4 as a housekeeping gene. *p<0.05.
(PDF)

S2 Fig The abundance of nephrin in rat cortex and outer medulla. Cortex and medullary material from 3-month old Wistar rats were immunoblotted for nephrin. Cm, Coomassie.
(PDF)

S3 Fig The effect of lithium on interstitial fibrosis in BTBR-ob/ob mice. 12-week old female BTBR-WT and -*ob/ob* mice received standard chow or chow with lithium supplementation (10 or 40 LiCl mmol/kg) for 12 weeks. After sacrifice, kidneys were isolated and the absence (A) or presence of minimal (B) or mild (C) interstitial fibrosis was determined using Picro Sirius Red staining and (D) scored with respective scores of 0, 1 or 2. Minimal interstitial fibrosis was defined as the presence of fibrotic tissue in up to 20% of whole kidney. When this presence exceeded 20%, but was less than 40%, it was defined as mild. In none of the kidneys the presence of interstitial fibrosis extended 40% of the whole area.
(PDF)

S4 Fig The effect of lithium on mRNA expression on various inflammatory markers. 12-week old female BTBR-WT and -*ob/ob* mice received standard chow or chow with lithium supplementation (10 or 40 LiCl/kg) for 12 weeks. After RNA isolation from cortex, mRNA levels of TNFα, IFNY, F4/80, CD68, MCP1 and IL-1RA were determined by qPCR using 36B4 as a housekeeping gene.
(PDF)

S5 Fig pGSK3/GSK3 ratio in BTBR-*ob/ob* mice. 12-week old female BTBR- *ob/ob* mice received standard chow (Ctr) or chow with lithium supplementation (10 or 40 LiCl/kg). After 12 weeks kidneys were isolated and protein lysates were immunoblotted for pGSK3 and GSK3. Representative immunoblots for *ob/ob* mice for (A) GSK3 and (B) pGSK3 are depicted. Corresponding densitometric analysis of (C) total GSK3 abundance and pGSK3/GSK3 ratio (n = 5–9 per group). Cm, Coomassie.
(PDF)

S1 Table Primer sequences. (PDF)

S2 Table Blood glucose levels 30, 60 and 120 minutes after intraperitoneal glucose injection. (PDF)
