# Supplementary material for: Lithium reduces blood glucose levels, but aggravates albuminuria in BTBR-ob/ob mice
Source: PLoS One. 2017 Dec 15;12(12):e0189485. doi: 10.1371/journal.pone.0189485 (PMC5731748; doi:10.1371/journal.pone.0189485)
Supplement: S1 Fig — 12-week old female BTBR-WT and -ob/ob mice received standard chow or chow with lithium supplementation (10 or 40 LiCl/kg) for 12 weeks. After RNA isolation from cortex, mRNA levels of nephrin, podocin, podoplanin and synaptopodin were determined by qPCR using 36B4 as a housekeeping gene. *p<0.05. (PDF) [file pone.0189485.s001.pdf]

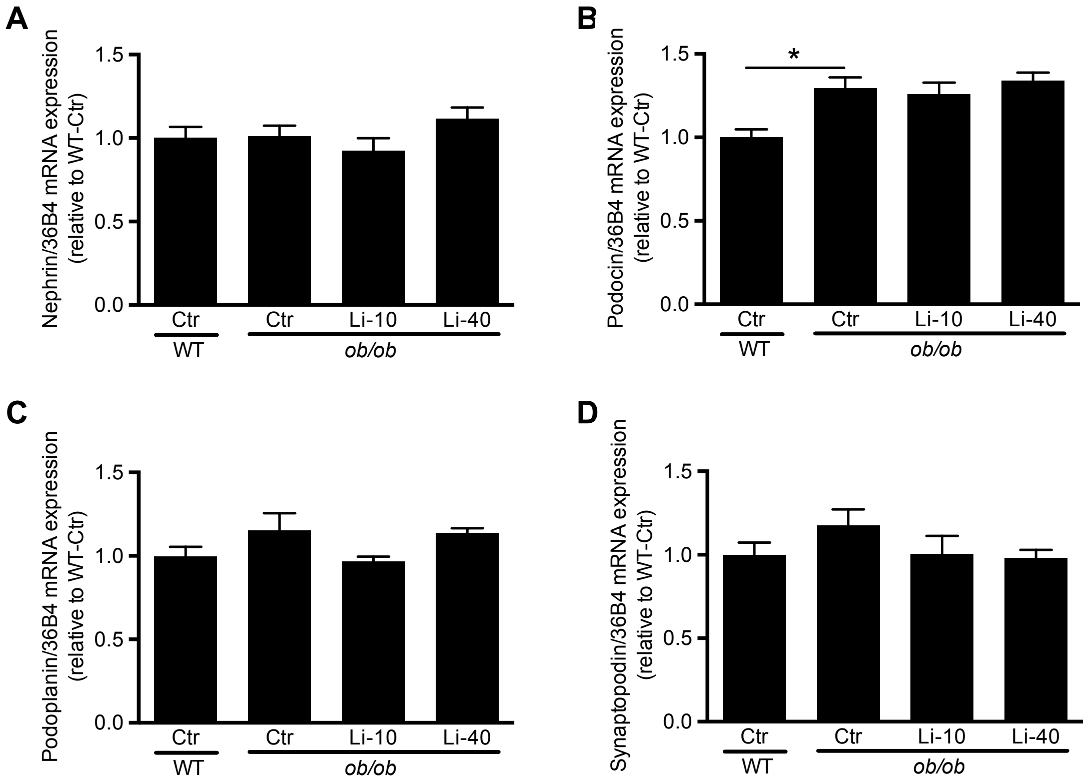

S1 Fig. The effect of lithium on mRNA levels of nephrin, podocin, podoplanin and synaptopodin. 12-week old female BTBR-WT and -*ob/ob* mice received standard chow or chow with lithium supplementation (10 or 40 LiCl/kg) for 12 weeks. After RNA isolation from cortex, mRNA levels of nephrin, podocin, podoplanin and synaptopodin were determined by qPCR using 36B4 as a housekeeping gene. *p<0.05.
