# Supplementary material for: Lithium reduces blood glucose levels, but aggravates albuminuria in BTBR-ob/ob mice
Source: PLoS One. 2017 Dec 15;12(12):e0189485. doi: 10.1371/journal.pone.0189485 (PMC5731748; doi:10.1371/journal.pone.0189485)
Supplement: S2 Fig — Cortex and medullary material from 3-month old Wistar rats were immunoblotted for nephrin. Cm, Coomassie. (PDF) [file pone.0189485.s002.pdf]

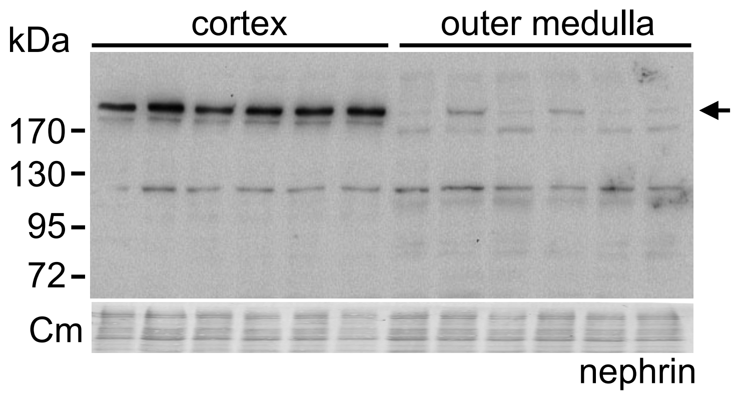

S2 Fig. The abundance of nephrin in rat cortex and outer medulla. Cortex and medullary material from 3-month old Wistar rats were immunoblotted for nephrin. Cm, Coomassie.
