# Supplementary material for: Lithium reduces blood glucose levels, but aggravates albuminuria in BTBR-ob/ob mice
Source: PLoS One. 2017 Dec 15;12(12):e0189485. doi: 10.1371/journal.pone.0189485 (PMC5731748; doi:10.1371/journal.pone.0189485)
Supplement: S3 Fig — 12-week old female BTBR-WT and -ob/ob mice received standard chow or chow with lithium supplementation (10 or 40 LiCl mmol/kg) for 12 weeks. After sacrifice, kidneys were isolated and the absence (A) or presence of minimal (B) or mild (C) interstitial fibrosis was determined using Picro Sirius Red staining and (D) scored with respective scores of 0, 1 or 2. Minimal interstitial fibrosis was defined as the presence of fibrotic tissue in up to 20% of whole kidney. When this presence exceeded 20%, but was less than 40%, it was defined as mild. In none of the kidneys the presence of interstitial fibrosis extended 40% of the whole area. (PDF) [file pone.0189485.s003.pdf]

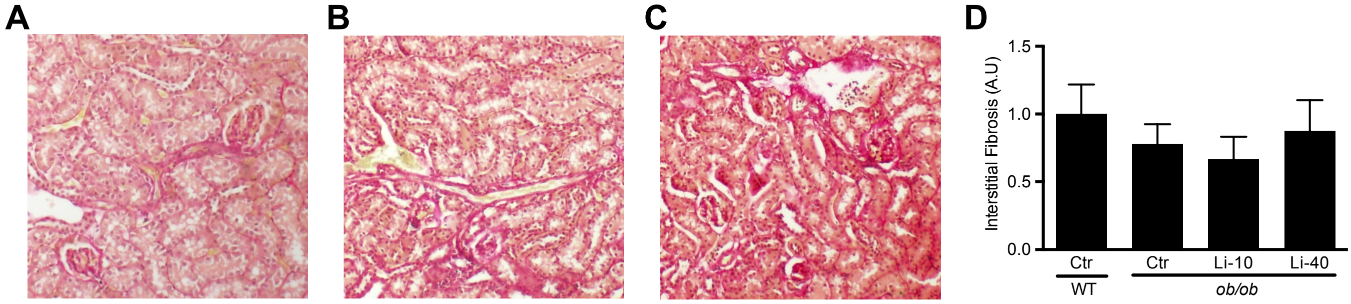

S3 Fig. The effect of lithium on interstitial fibrosis in BTBR-ob/ob mice. 12-week old female BTBR-WT and -*ob/ob* mice received standard chow or chow with lithium supplementation (10 or 40 LiCl mmol/kg) for 12 weeks. After sacrifice, kidneys were isolated and the absence (A) or presence of minimal (B) or mild (C) interstitial fibrosis was determined using Picro Sirius Red staining and (D) scored with respective scores of 0, 1 or 2. Minimal interstitial fibrosis was defined as the presence of fibrotic tissue in up to 20% of whole kidney. When this presence exceeded 20%, but was less than 40%, it was defined as mild. In none of the kidneys the presence of interstitial fibrosis extended 40% of the whole area.
