# Supplementary material for: Lithium reduces blood glucose levels, but aggravates albuminuria in BTBR-ob/ob mice
Source: PLoS One. 2017 Dec 15;12(12):e0189485. doi: 10.1371/journal.pone.0189485 (PMC5731748; doi:10.1371/journal.pone.0189485)
Supplement: S5 Fig — 12-week old female BTBR- ob/ob mice received standard chow (Ctr) or chow with lithium supplementation (10 or 40 LiCl/kg). After 12 weeks kidneys were isolated and protein lysates were immunoblotted for pGSK3 and GSK3. Representative immunoblots for ob/ob mice for (A) GSK3 and (B) pGSK3 are depicted. Corresponding densitometric analysis of (C) total GSK3 abundance and pGSK3/GSK3 ratio (n = 5–9 per group). Cm, Coomassie. (PDF) [file pone.0189485.s005.pdf]

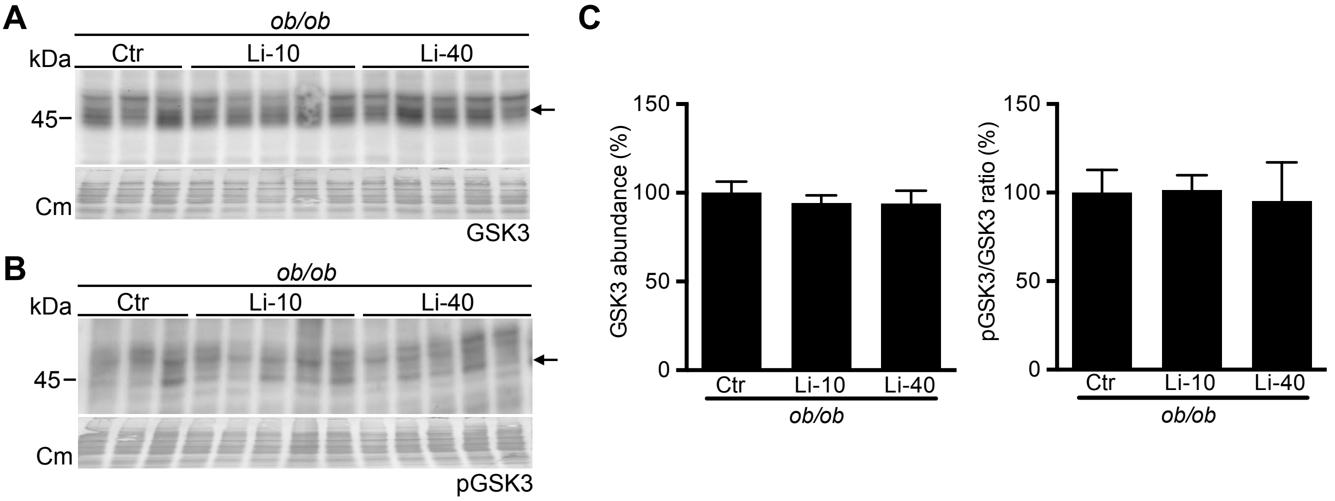

S5 Fig. pGSK3/GSK3 ratio in BTBR-*ob/ob* mice. 12-week old female BTBR- *ob/ob* mice received standard chow (Ctr) or chow with lithium supplementation (10 or 40 LiCl/kg). After 12 weeks kidneys were isolated and protein lysates were immunoblotted for pGSK3 and GSK3. Representative immunoblots for *ob/ob* mice for (A) GSK3 and (B) pGSK3 are depicted. Corresponding densitometric analysis of (C) total GSK3 abundance and pGSK3/GSK3 ratio (n=5-9 per group). Cm, Coomassie.
