# Supplementary material for: Lithium reduces blood glucose levels, but aggravates albuminuria in BTBR-ob/ob mice
Source: PLoS One. 2017 Dec 15;12(12):e0189485. doi: 10.1371/journal.pone.0189485 (PMC5731748; doi:10.1371/journal.pone.0189485)
Supplement: S1 Table — (PDF) [file pone.0189485.s006.pdf]

S1 Table. Primer sequences.

Gene	Protein	Fw primer 5' > 3'	Rv primer 5' > 3'	Melting Temp
Rplp0	36B4	AGCGCGTCCTGGCATTGTGTGG	GGGCAGCAGTGGTGGCAGCAGC	59°C
Nphs1	Nephrin	GTGCCCTGAAGGACCCTAC	CCTGTGGATCCCTTTGACAT	59°C
Nphs2	Podocin	CTTGGCACATCGATCCCTC	GCACTTTGGCCTGTCTTTG	59°C
Pdpn	Podoplanin	CACCTCAGCAACCTCAGAC	GTTTCATCCCCTGCATTATC	59°C
Synpo	Synaptopodin	CGGACCTTCTTCCTGTGCTA	ATCCTCGGAGTCGAGAGACA	59°C
Tnf	TNFα	CATCTTCTCAAAATTCGAGTGACAA	TGGGAGTAGACAACGTACAACCC	59°C
Ifng	IFNγ	GGTGACCTTGTGACAAGCTC	TGCTGTGTGGTCTGTCTGTC	59°C
Adgre1	F4/80	CTTTGGCTATGGGCTTCCAGTC	GCAAGGAGGACAGAGTTTATCGTG	59°C
Cd68	CD68	CCAATTCAGGGTGGAAGAAA	CTCGGGCTCTGATGTAGGTC	59°C
Ccl2	MCP1	CCCAATGAGTAGGCTGGAGA	TCTGGACCCATTCCTTCTTG	59°C
Il1rn	IL-1RA	AAATCTGCTGGGGACCCTAC	TGAGCTGGTTGTTTCTCAGG	59°C
Acta2	αSMA	TGTGCTGGACTCTGGAGATG	ATGTCACGGACAATCTCACG	59°C
Ctgf	CTGF	GTCCAGACCACAGAGTGGAG	CTCCAGGTCAGCTTCGCAG	59°C
Tgfb1	TGFβ1	CTGGAGTTGTACGGCAGTGG	TGGGGCTGATCCCGTTGA	59°C
