# Supplementary material for: Lithium reduces blood glucose levels, but aggravates albuminuria in BTBR-ob/ob mice
Source: PLoS One. 2017 Dec 15;12(12):e0189485. doi: 10.1371/journal.pone.0189485 (PMC5731748; doi:10.1371/journal.pone.0189485)
Supplement: S2 Table — (PDF) [file pone.0189485.s007.pdf]

S2 Table. Blood glucose levels 30, 60 and 120 minutes after intraperitoneal glucose injection.

	**WT-Ctr**			***ob/ob*-Ctr**			***ob/ob*-Li-10**			***ob/ob*-Li-40**		
**Mouse:**	**30**	**60**	**120**	**30**	**60**	**120**	**30**	**60**	**120**	**30**	**60**	**120**
1	431	248	156	>600	>600	552	>600	>600	554	496	>600	419
2	235	192	151	>600	>600	488	>600	>600	>600	>600	>600	>600
3	214	172	139	>600	>600	>600	476	565	468	>600	>600	581
4	192	189	154	>600	>600	>600	548	557	286	580	509	>600
5	218	151	102	>600	>600	>600	565	>600	522	561	512	574
6	232	179	114	585	>600	581	494	>600	439	526	533	402
7	248	193	143	545	594	330	>600	>600	486	422	454	436
8	231	175	120	548	541	570	480	421	215	>600	>600	>600
9	232	179	145	>600	>600	504	>600	>600	576	441	512	395
